# Effectiveness of Different Methods for Baroreflex Sensitivity Assessment in Determining the Severity of Cardiovascular Autonomic Neuropathy in Patients With Parkinson’s Disease

**DOI:** 10.3389/fnins.2022.833344

**Published:** 2022-02-25

**Authors:** Chih-Cheng Huang, Yun-Ru Lai, Chia-Yi Lien, Ben-Chung Cheng, Chia-Te Kung, Yi-Fang Chiang, Cheng-Hsien Lu

**Affiliations:** ^1^Department of Neurology, Kaohsiung Chang Gung Memorial Hospital, Chang Gung University College of Medicine, Kaohsiung, Taiwan; ^2^Department of Internal Medicine, Kaohsiung Chang Gung Memorial Hospital, Chang Gung University College of Medicine, Kaohsiung, Taiwan; ^3^Department of Emergency Medicine, Kaohsiung Chang Gung Memorial Hospital, Chang Gung University College of Medicine, Kaohsiung, Taiwan; ^4^Center for Shockwave Medicine and Tissue Engineering, Kaohsiung Chang Gung Memorial Hospital, Chang Gung University College of Medicine, Kaohsiung, Taiwan; ^5^Department of Biological Science, National Sun Yat-sen University, Kaohsiung, Taiwan; ^6^Department of Neurology, Xiamen Chang Gung Memorial Hospital, Xiamen, China

**Keywords:** baroreflex sensitivity, Valsalva maneuver, sequence method, spectral method, Parkinson’s Disease

## Abstract

**Background:**

Autonomic disorders are an important non-motor feature of Parkinson’s disease (PD). Baroreflex sensitivity (BRS) is often used as an indicator of cardiovascular autonomic function, and it is clinically significant. Several different methods of BRS assessment have been described. We evaluated and compared the efficiency of several methods of BRS assessment for additional insight into the underlying physiology and the determination of its severity in patients with PD.

**Materials and Methods:**

Eighty-five patients with PD underwent cardiovascular autonomic testing. The Composite Autonomic Scoring Scale (CASS) was used to grade the severity of autonomic impairment and to define the presence of cardiovascular autonomic neuropathy (CAN). BRS was assessed using the Valsalva maneuver (BRS_VM). In addition, spontaneous BRS was computed using the sequence method and the spectral method.

**Results and Conclusion:**

There was considerable agreement between the different methods of BRS assessment. Nevertheless, BRS_VM exhibited a higher degree of correlation with cardiovascular autonomic function than spontaneous BRS indexes obtained by the sequence or spectral method. BRS_VM, rather than spontaneous BRS, also had a predictive value for the presence of CAN to the diagnostic criteria by CASS in patients with PD.

## Introduction

Autonomic disorders have been recognized as an important non-motor feature of Parkinson’s disease (PD) (Martinez-Martin et al., 2011; Kim et al., 2014). Several studies have demonstrated that patients with PD exhibit decreased baroreflex sensitivity (BRS) (Szili-Torok et al., 2001; Blaho et al., 2017). The pattern of autonomic impairment provides an important clue to differentiate patients with PD from those with multiple system atrophy (Kimpinski et al., 2012; Pavy-LeTraon et al., 2018). In patients with severe autonomic failure, adrenergic impairment and decreased BRS cause orthostatic hypotension, which has a considerable impact on the patient’s quality of life. Furthermore, patients with PD have recently been found to have an increased risk of cardiovascular or cerebrovascular events (Alves et al., 2020; Park et al., 2020). The decreased BRS noted in patients with PD may be a contributing factor for the increased cardiovascular risk.

Baroreflex sensitivity (BRS) in humans can be assessed using several different methods (Parati et al., 2000). Generally, these methods are divided into two categories: In the first category, BRS is assessed in a laboratory setting using an external stimulus that triggers a change in the blood pressure (BP) and a subsequent change in the heart rate (HR). In the second category, BRS is determined using spontaneous oscillations of BP and HR without external interventions. There is no “gold standard” method for BRS evaluation, as different methods may describe different aspects of baroreflex modulation. Nevertheless, certain methods may be especially suitable under specific circumstances or can be more clinically significant than others for a particular group of patients.

There is a paucity of studies regarding the comparison of various methods of BRS assessment in patients with PD. This study aimed to evaluate BRS in patients with PD using three different methods and to compare the clinical relevance of each method. Our hypothesis is that it may provide additional insight into the underlying physiology to evaluate the diversity of various BRS assessments. Therefore, an optimal assessment can be chosen in a specific clinical context or for a certain group of patients.

## Patients and Methods

### Study Design and Patient Selection

We prospectively evaluated patients with a definitive diagnosis of idiopathic PD according to the clinical diagnostic criteria and magnetic resonance image findings (Hughes et al., 1992; Heim et al., 2017). The exclusion criteria were as follows: (1) cognitive impairment leading to inability to follow our instructions; (2) a known history of cardiovascular or cerebrovascular events; (3) presence of cardiovascular autonomic neuropathy related to diabetes or other etiologies; and (4) presence of an implanted pacemaker or any type of arrhythmia that prevented BRS assessment. The Institutional Review Committee on Human Research of the hospital approved this study (IRB 201901802B0). All participants received verbal and written information about the purpose of the study and signed informed consent forms.

### Clinical Assessment of Parkinson’s Disease

The clinical assessments of PD were performed using the Hoehn and Yahr Scale (Hoehn and Yahr, 1967) and the Unified Parkinson’s Disease Rating Scale (UPDRS) (Martinez-Martin et al., 1994), and took place during the “off” state, which was defined as 12 h or more after administration of the last dose of antiparkinsonian therapy. The age at disease onset, sex, body height, body weight, body mass index (BMI), disease duration, and levodopa dosage [expressed as Levodopa Equivalent Dose (LED)] (Tomlinson et al., 2010) were recorded for all participants. The autonomic symptoms profile of each patient was assessed using the Composite Autonomic Symptom Score 31 (COMPASS 31) questionnaire (Sletten et al., 2012), and the cognitive function was evaluated using the Cognitive Abilities Screening Instrument (CASI C v2.0).

### Autonomic Function Testing

All participants underwent a standardized evaluation of cardiovascular autonomic function during the “off” state, including heart rate response to deep breathing (HRDB), the Valsalva maneuver (VM), and the head-up tilt test (Low, 2003). In addition, 5 min of resting ECG recording and continuous BP monitoring were performed in the interval between the VM and the head-up tilt test. To avoid the influence of depth and frequency of breathing on spontaneous BRS, the patients had 10 min of rest between the end of VM and the 5-min recording of HR and BP. As for the number of VM testing, we followed the standardization of autonomic testing recommended by Low (2003); VM was repeated until 2 reproducible responses were obtained. The mean value of BRS_VM from these repeated procedures was registered. To avoid potential variability due to circadian rhythms, all tests were performed between 9:00 a.m. and 12:00 p.m. Patients receiving medications known to cause orthostatic hypotension or otherwise affect testing results were asked to stop drug treatment for a period corresponding to five half-lives before testing, provided that it was not detrimental to their wellbeing.

The HR was recorded continuously using a standard three-lead ECG monitor (Ivy Biomedical, model 3000; Branford, CT, United States), and the BP was measured continuously using beat-to-beat photoplethysmographic recording (Finameter Pro, Ohmeda; Englewood, OH, United States). The Valsalva ratio (VR) and HRDB parameters were obtained using the WR Testworks software (WR Medical Electronics Company, Stillwater, MN, United States) and calculated as described by Low (2003). The severity of the patient’s cardiovascular autonomic impairment was graded using the Composite Autonomic Scoring Scale (CASS) (Low, 1993). The patients were considered to meet the definition of cardiovascular autonomic neuropathy (CAN) if they exhibited a minimum CASS score of one in both the cardiovagal and the adrenergic domains or a minimum score of two in a single domain; that is, when the CASS score was equal to or higher than two.

### Baroreflex Sensitivity Assessment

The BRS was assessed using three different methods: the Valsalva maneuver (BRS_VM) in the laboratory, and the spontaneous BRS by sequence and spectral methods. BRS_VM was calculated by least-squares regression analysis from changes in HR and BP during the early phase II of the VM. In contrast, spontaneous BRS was computed from BP and HR records at rest, and then the BRS_seq and α-index (both at low frequency, α-LF, and in high frequency, α-HF) were obtained. The computation was performed using the Nevrokard™ BRS software package (Nevrokard, Slovenia) according to a previously described criteria to estimate BRS_seq (Huang et al., 2020). For the spectral method, the oscillations of systolic BP and the RR interval were transformed to the frequency domain using fast Fourier transform. The spectral powers were divided into two frequency bands: LF (0.04–0.15 Hz) and HF (0.15–0.4 Hz). The BRS α-index was computed as the mean of the square roots of the ratios of the spectral powers of RRI to SBP in the LF and HF ranges (α-LF and α-HF, respectively) if the coherence between these two signals was > 0.5.

### Statistical Analysis

Data are expressed as mean ± SD or median (interquartile range, IQR) for continuous variables and as median (IQR) for ordinal variables. Associations between the measurements were evaluated using the Pearson correlation test for normally distributed continuous variables or by the Spearman non-parametric test for continuous variables with skewness or ordinal variables. Furthermore, receiver operating characteristic (ROC) curves were generated for the BRS indexes that showed significant differences between the experimental groups for predicting the presence of CAN. The threshold for statistical significance was set at *p* < 0.05. All statistical analyses were conducted using IBM SPSS Statistics v23 statistical software (IBM, Redmond, WA, United States).

## Results

### Demographic Data of Patients With Parkinson’s Disease

The 85 patients recruited in this study included 42 men and 43 women. Of the 85 patients, ten exhibited suboptimal effort in the performance of VM, which prevented the computation of a valid CASS score. Of the remaining 75 patients, 35 had CAN and 40 did not. The demographic data per group are listed in Table 1. Their demographic data, as well as their functional status (UPDRS), LED, and medication information, are listed in Table 1. In addition, 22 age- and sex-matched subjects (11 men and 11 women) were selected from the control database of our autonomic laboratory. Their autonomic parameters are listed in Table 2 as normal reference of our laboratory.

**TABLE 1:** Baseline characteristics of enrolled subjects and controls.

	Controls (*n* = 22)	PD patients (*n* = 85)*		
		
		CAN (*n* = 35)	Non-CAN (*n* = 40)	Total (*n* = 85)
Age, years	66.6 ± 7.8	68.0 ± 8.7	63.3 ± 9.6	67.5 ± 9.7
Sex (men/women)	11/11	16/19	19/21	42/43
Height (m)	1.60 ± 0.10	1.58 ± 0.08	1.59 ± 0.07	1.59 ± 0.07
Body weight (kg)	63.0 ± 13.4	63.4 ± 10.4	62.9 ± 12.3	63.4 ± 11.3
Body mass index (kg/m^2^)	24.3 ± 3.5	25.4 ± 4.2	24.7 ± 4.5	25.0 ± 4.2
Disease duration, years	–	6.0 ± 4.4	5.8 ± 4.8	5.5 ± 4.4
LED (mg/day)	–	918.3 ± 585.6	799.4 ± 600.1	828.4 ± 575.7
UPDRS total score^α^	–	31 [23, 37]	22.5 [15, 35.3]	27 [18, 37.5]
UPDRS I ^β^	–	2 [1, 3]	1 [0, 2]	2 [1, 3]
UPDRS II (ADL score) ^γ^	–	11 [7, 14]	7.5 [5, 13.8]	10 [5, 13]
UPDRS III (motor score) ^δ^	–	18 [14, 25]	13.5 [9, 19]	16 [10.5, 22]
Cognitive abilities screening instrument	–	78.7 ± 17.5	89.3 ± 7.1	83.6 ± 13.9
Total weighted COMPASS 31 score	–	19.7 ± 11.8	14.0 ± 9.3	16.1 ± 10.6
Anti-Parkinsonian medications^Φ^				
Levodopa	–	34	34	75
Dopamine agonist (Pramipexole/Ropinirole)	–	23	30	56
MAO-B inhibitors (Selegiline/Rasagiline)	–	12	14	30
COMT inhibitors (Entacapone)	–	5	6	12
Amantadine	–	4	4	8

*Ten of the patients were unclassified due to a lack of a valid score of Composite Autonomic Scoring Scale. Φ = All the patients took more than one kind of anti-Parkinsonian medications.

*CAN, cardiovascular autonomic neuropathy; UPDRS, Unified Parkinson’s Disease Rating Scale; LED, Levodopa equivalent dose, MAO-B, monoamine oxidase B, COMT, catechol-o-methyl-transferase; COMPASS, Composite Autonomic Symptom Scale.*

*α = “Total UPDRS” score is the combined sum of parts I, II, and III. β = I. Mentation, behavior, and mood. γ = II. Activities of daily living (ADL). δ = III. Motor examination.*

**TABLE 2:** Comparison of parameters of autonomic function and composite autonomic symptom scale 31 between PD with or without CAN.

	Normal reference (*n* = 22)	PD patients (*n* = 75)		
		
		CAN (*n* = 35)	Non-CAN (*n* = 40)	*p*-value
**Composite autonomic symptom scale 31**				
Orthostatic intolerance	0	1.7 ± 1.2	0.6 ± 0.4	0.01*
Vasomotor score	0	0.7 ± 0.3	0.7 ± 0.6	0.72
Secretomotor score	0	2.1 ± 1.5	2.0 ± 1.6	0.64
Gastrointestinal symptoms score	0	5.5 ± 3.5	5.1 ± 3.6	0.64
Bladder score	0	2.0 ± 1.7	1.3 ± 1.2	0.02*
Pupillomotor score	0	3.7 ± 2.7	4.5 ± 2.6	0.22
Total weighted COMPASS score	0	19.7 ± 11.8	14.0 ± 9.3	0.02*
**Cardiovascular autonomic function**				
Composite autonomic symptom scale	0	2.7 ± 1.0	0.5 ± 0.3	< 0.0001*
Adrenergic subscore	0	1.0 ± 0.8	0.5 ± 0.3	< 0.0001*
Cardiovagal subscore	0	1.7 ± 0.9	0.4 ± 0.2	< 0.0001*
Heart rate response to deep breathing (beats/min)	11.0 ± 4.6	5.1 ± 1.8	10.4 ± 4.9	< 0.0001*
Valsalva ratio	1.5 ± 0.2	1.3 ± 0.3	1.4 ± 0.2	0.02*
BP drop during head-up tilt (mmHg)	2.2 (–2.8, 9.5)	11.0 (3.0, 24.0)	5.0 (-1.0, 12.8)	0.08
**Baroreflex sensitivity methods**				
BRS_VM (ms/mmHg)	2.3 ± 1.5	1.3 ± 0.8	2.2 ± 1.3	0.001*
BRS_Seq (ms/mmHg)	8.0 ± 4.0	5.7 ± 2.6	6.5 ± 3.0	0.2
a-LF (ms/mmHg)	8.5 ± 4.5	8.4 ± 7.9	7.7 ± 4.6	0.72
α-HF (ms/mmHg)	11.0 ± 6.9	7.8 ± 5.5	12.1 ± 9.4	0.09

Values are expressed as mean ± SD or median [interquartile range (IQR)], *p < 0.05 (the comparison is between groups of CAN and non-CAN).

*CAN, cardiovascular autonomic neuropathy; BP, blood pressure; BRS_VM, baroreflex sensitivity obtained by Valsalva maneuver; BRS_seq, baroreflex sensitivity obtained by sequence method; α-LF, α index in low frequency; α-HF, α index in high frequency.*

### Comparison Between Patients With and Without Cardiovascular Autonomic Neuropathy

Table 2 shows the values for autonomic symptom profile (COMPASS 31 score), cardiovascular autonomic function (autonomic parameters and CASS score), and BRS indexes calculated by the different methods in patients with and without CAN. Among the COMPASS 31 scores, the orthostatic intolerance, bladder, and total weighted scores were significantly different between the two groups. All cardiovascular autonomic parameters and the CASS score, as well as the subscores, showed a significant difference between the two groups with the exception of BP change during head-up tilt, which appeared to be higher in the CAN group but did not reach statistical significance. Regarding the BRS indexes, significant differences between the groups were only observed in BRS_VM. Neither of the autonomic parameters showed significant difference when comparing the group of non-CAN with normal reference, but all of the autonomic parameters except the two α-indexes (α-LF and α-HF) showed significant difference when comparing the group of CAN with normal reference. (The statistical results of the comparison with normal reference are not listed in Table 2).

### Correlation Analysis Between Composite Autonomic Scoring Scale and the Different Baroreflex Sensitivity Indexes

**FIGURE 1:**
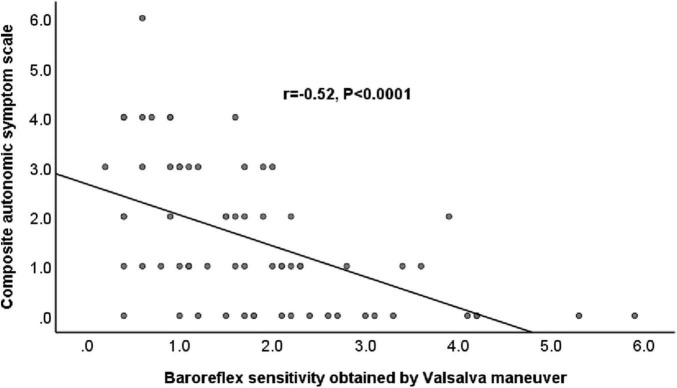
Relationship between baroreflex sensitivity obtained by Valsalva maneuver and Composite Autonomic Scoring Scale in patients with Parkinson’s disease.

Table 3 shows the results of the correlations between CASS and the BRS indexes. BRS_VM exhibited significant correlations with the CASS score and with the subscores in both cardiovagal and adrenergic domains (Figure 1). BRS_seq exhibited significant correlations with the CASS score and cardiovagal subscore, but not with the adrenergic subscore. There was no significant correlation between the α-index and the CASS score. The correlation analysis also evaluated the relationship between each BRS index. The correlation between BRS_VM and α-LF was not significant. There were significant correlations among all the other BRS indexes.

**TABLE 3:** Correlation analysis between CASS and different BRS indexes.

Spearman correlation	BRS_VM		BRS_Seq		a-LF		a-HF	
				
	*r*	*p*	*r*	*p*	*r*	*p*	*r*	*p*
Composite autonomic scoring scale	–0.52	< 0.0001*	–0.28	0.02*	–0.06	0.72	–0.37	0.01*
Adrenergic sub score	–0.41	0.001*	–0.11	0.34	–0.01	0.94	–0.24	0.12
Cardiovagal sub score	–0.43	< 0.0001*	–0.30	0.01*	–0.10	0.52	–0.35	0.02*
Different BRS indexes								
BRS_VM	–	–	0.279	0.024*	0.033	0.840	0.424	0.007*
BRS_seq			–	–	0.571	< 0.001*	0.936	< 0.001*
α-LF					–	–	0.492	0.001*
α-HF							–	–

* Indicates that p-value < 0.05.

*CASS, Composite autonomic scoring scale; BRS, baroreflex sensitivity; BRS_VM, baroreflex sensitivity obtained by Valsalva maneuver; BRS_seq, baroreflex sensitivity obtained by sequence method; α-LF, α index in low frequency; α-HF, α index in high frequency.*

### Diagnostic Accuracy for Cardiovascular Autonomic Neuropathy Using Receiver Operating Characteristic Curve Analysis

**FIGURE 2:**
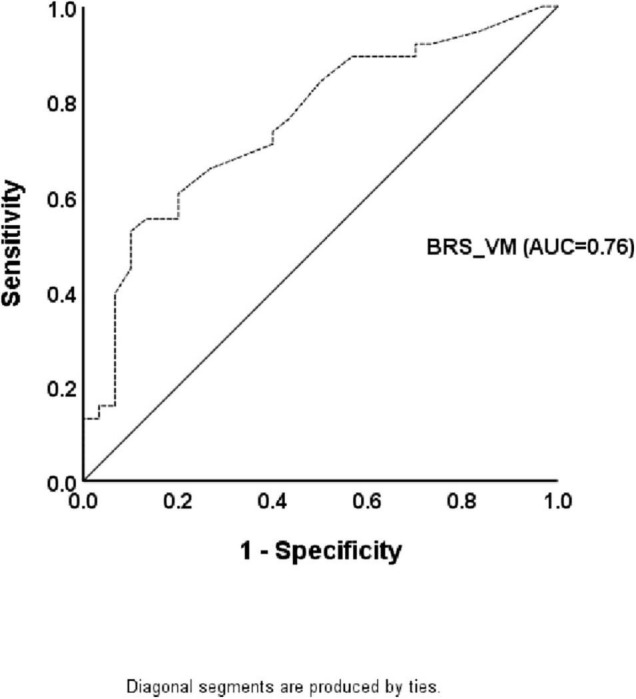
The receiver operating characteristic curve for the presence of cardiovascular autonomic neuropathy in patients with Parkinson’s disease. The diagnostic accuracy of baroreflex sensitivity obtained by the Valsalva maneuver is shown based on the receiver operating characteristic curve analysis.

The significant statistical analyses for predicting the presence of CAN using the ROC curve analysis are listed in Table 4. Only BRS_VM showed diagnostic accuracy for the presence of CAN (*p* < 0.05). The cutoff value for the presence of CAN in patients with PD was 1.25 (AUC = 0.76, *p* < 0.0001), and the sensitivity and specificity were 74% and 60%, respectively (Figure 2 and Table 4).

**TABLE 4:** Sensitivity, specificity, and area under the curve using receiver operating characteristic curve analysis for baroreflex sensitivity obtained by Valsalva maneuver in predicting cardiovascular autonomic neuropathy.

Significant parameters	Cutoff value	AUC (95% CI)	Sensitivity (%)	Specificity (%)	*p*-value
BRS_VM	1.25	0.76 (0.64–0.87)	74	60	< 0.0001*

*p < 0.01; ROC, receiver operating characteristic; BRS_VM, baroreflex sensitivity obtained by Valsalva maneuver; AUC, area under the curve.

## Discussion

### Major Findings

Despite the remarkable agreement between the different methods for BRS assessment, BRS_VM had stronger correlation with CASS, which represents the severity of autonomic impairment, compared to spontaneous BRS indexes. BRS_VM also had a higher predictive value for the presence of CAN according to the diagnostic criteria by CASS in patients with PD than spontaneous BRS indexes.

### Comparison of Different Baroreflex Sensitivity Assessments

To estimate BRS, the VM method explores the arterial baroreflex modulation of the heart through the quantification of tachycardia that occurs during the initial decrease in the BP at early phase II. An alternative method uses bradycardia during the subsequent increase in BP after the cessation of expiratory pressure (phase IV) (Goldstein et al., 1982). The former was selected because phase IV can be absent in patients with autonomic impairment such as the ones recruited for the present study (Sandroni et al., 1991). The disadvantage of BRS_VM is that the VM also triggers alterations in the chemoreceptor and cardiopulmonary receptor activity, which makes HR responses less specific. Specificity is also reduced by the concomitant stimulation of skeletal muscle receptors due to increased muscle tone during VM (Parati et al., 2000). Finally, active cooperation of the subjects being tested is required. This should be particularly taken into account in patients with PD, a certain proportion of whom fail to achieve the required effort due to motor or pulmonary dysfunction. This was the reason we did not manage to obtain valid CASS and BRS_VM data from ten of the patients that we had originally recruited. Spontaneous BRS techniques do not require any external intervention on the subject being tested, thus avoiding the aforementioned limitations. However, they still present some disadvantages. The sequence method is of limited use in patients with autonomic dysfunction such as the ones that participated in the present study because diminished BP fluctuations lead to a lack of a significant correlation with changes in the RR interval (Oka et al., 2003). The α-index is also unsuitable for these patients because of the small coherence between BP and HR (< 0.5). Our data showed no significant correlation between the α-index and the CASS score (Table 3), but the correlation was actually significant between α-HF and the CASS score in the subgroup of non-CAN (Spearman’s rho = –0.550, *p* = 0.005). In other words, the correlation between CASS and α-HF was disrupted due to the existence of CAN. To sum up, spontaneous BRS may not be suitable to be used in patients with known autonomic impairment, such as the PD patients in this study.

There was a significant correlation between each of the BRS measures obtained by different techniques, but no correlation between BRS_VM and α-LF was identified. α-LF is influenced by additional factors such as Mayer waves in addition to baroreflex modulation (Julien, 2006; Silva et al., 2019). In subjects with intact autonomic function, baroreflex modulation is the key factor to determine α-LF. However, the influence from other factors becomes prominent in patients having autonomic impairment with reduced baroreflex modulation, such as the PD patients in our study. The reason may explain the lack of correlation between α-LF and BRS_VM in our patients. In contrast to our results that showed a significant correlation between BRS_VM and spontaneous BRS (except α-LF), the study by Yang et al. revealed a positive association between spontaneous sympathetic BRS and VM sympathetic BRS, but no correlation between spontaneous and VM cardiovagal BRS (Yang and Carter, 2013). We did not measure sympathetic BRS in the current study. The inconsistency in cardiovagal BRS may be due to different ages (much younger in Yang’s study) and different characters (healthy subjects vs. patients with PD) of study subjects. Various BRS estimates are not interchangeable. A finding noted in a certain study cannot be applied to subjects with different context.

### Baroreflex Sensitivity in Patients With Parkinson’s Disease

Blunted BRS has been noted in previous reports (Szili-Torok et al., 2001; Blaho et al., 2017). Our data revealed that reduced BRS was noted in the CAN group but not in the non-CAN group. Furthermore, the significant difference was exhibited in BRS_VM and BRS_seq, but not in α-LF or α-HF. The finding supports the aforementioned notion that α-index is unsuitable to be used in these PD patients.

### Study Limitations

There are some limitations to our study. First, we only had the database of the control group from our autonomic laboratory as normal reference for comparison. Although the case number was only 22, our normal reference of spontaneous BRS is similar to the one reported by Tank et al. (2000). Second, the study recruited only patients with comparatively better functions at a relatively early stage. We are uncertain whether our results can be applied to patients in advanced stages of PD. In addition, we only had early phase II for computing BRS_VM, and thus only tachycardia to BP decrease was assessed in the present study. BRS_VM can also be computed in phase IV to assess the function of bradycardia response to BP increase. As mentioned above, however, it was not done in the study because a certain part of our enrolled patients had absent phase IV due to adrenergic impairment. Finally, only three assessment methods for BRS were used in this study due to the laboratory settings and software availability. Further studies that include additional methods are required to comprehensively elucidate baroreflex modulation.

## Conclusion

Our results showed considerable agreement between different methods for BRS assessment. Among them, BRS_VM had stronger correlation with CASS and was the only method that had a significant predictive value for the presence of CAN in patients with PD.

## Data Availability Statement

The raw data supporting the conclusions of this article will be made available by the authors, without undue reservation.

## Ethics Statement

This study was conducted according to the guidelines of the Declaration of Helsinki, and approved by the Institutional Review Board of Chang Gung Medical Foundation (IRB 201901802B0). The patients/participants provided their written informed consent to participate in this study.

## Author Contributions

C-CH and C-HL conceptualization, formal analysis, and writing—review and editing. C-CH, Y-RL, and C-HL methodology. C-CH, Y-RL, C-YL, B-CC, C-TK, Y-FC, and C-HL investigation. C-CH writing—original draft preparation. C-HL resources, supervision, project administration, and funding acquisition. All authors have read and agreed to the published version of the manuscript.

## Conflict of Interest

The authors declare that the research was conducted in the absence of any commercial or financial relationships that could be construed as a potential conflict of interest.

## Publisher’s Note

All claims expressed in this article are solely those of the authors and do not necessarily represent those of their affiliated organizations, or those of the publisher, the editors and the reviewers. Any product that may be evaluated in this article, or claim that may be made by its manufacturer, is not guaranteed or endorsed by the publisher.
